# Multilocus phylogeography of the common lizard *Zootoca vivipara* at the Ibero-Pyrenean suture zone reveals lowland barriers and high-elevation introgression

**DOI:** 10.1186/1471-2148-13-192

**Published:** 2013-09-10

**Authors:** Borja Milá, Yann Surget-Groba, Benoît Heulin, Alberto Gosá, Patrick S Fitze

**Affiliations:** 1 National Museum of Natural Sciences, Spanish Research Council (CSIC), José Gutiérrez Abascal 2, Madrid 28006, Spain; 2 Key Laboratory of Tropical Forest Ecology, Xishuangbanna Tropical Botanical Garden, Chinese Academy of Sciences, Menglun, Mengla, Yunnan 666303, P. R China; 3 Station Biologique, CNRS UMR 6553, Paimpont 35380, France; 4 Herpetology Department, Sociedad de Ciencias Aranzadi, San Sebastián 20014, Spain; 5 ARAID Foundation, Zaragoza 50004, Spain; 6 Instituto Pirenaico de Ecología (IPE-CSIC), Jaca 22700, Spain; 7 Université de Lausanne, Department of Ecology and Evolution (DEE), Biophore, Lausanne 1015, Switzerland

**Keywords:** Cytonuclear incongruence, Gene flow, Phylogeography, Secondary contact, Speciation, Vicariance

## Abstract

**Background:**

The geographic distribution of evolutionary lineages and the patterns of gene flow upon secondary contact provide insight into the process of divergence and speciation. We explore the evolutionary history of the common lizard *Zootoca vivipara* (= *Lacerta vivipara*) in the Iberian Peninsula and test the role of the Pyrenees and the Cantabrian Mountains in restricting gene flow and driving lineage isolation and divergence. We also assess patterns of introgression among lineages upon secondary contact, and test for the role of high-elevation trans-mountain colonisations in explaining spatial patterns of genetic diversity. We use mtDNA sequence data and genome-wide AFLP loci to reconstruct phylogenetic relationships among lineages, and measure genetic structure.

**Results:**

The main genetic split in mtDNA corresponds generally to the French and Spanish sides of the Pyrenees as previously reported, in contrast to genome-wide AFLP data, which show a major division between NW Spain and the rest. Both types of markers support the existence of four distinct and geographically congruent genetic groups, which are consistent with major topographic barriers. Both datasets reveal the presence of three independent contact zones between lineages in the Pyrenean region, one in the Basque lowlands, one in the low-elevation mountains of the western Pyrenees, and one in the French side of the central Pyrenees. The latter shows genetic evidence of a recent, high-altitude trans-Pyrenean incursion from Spain into France.

**Conclusions:**

The distribution and age of major lineages is consistent with a Pleistocene origin and a role for both the Pyrenees and the Cantabrian Mountains in driving isolation and differentiation of *Z. vivipara* lineages at large geographic scales. However, mountain ranges are not always effective barriers to dispersal, and have not prevented a recent high-elevation trans-Pyrenean incursion that has led to asymmetrical introgression among divergent lineages. Cytonuclear discordance in patterns of genetic structure and introgression at contact zones suggests selection may be involved at various scales. Suture zones are important areas for the study of lineage formation and speciation, and our results show that biogeographic barriers can yield markedly different phylogeographic patterns in different vertebrate and invertebrate taxa.

## Background

The geographical distribution of intraspecific lineages and the pattern of gene flow among them provides valuable insight into the process of lineage divergence and speciation [1]. Of particular interest are phylogeographic studies at large spatial scales that encompass major geographic barriers to gene flow as well as areas where divergent lineages come into secondary contact. Patterns of introgression at these contact zones can shed light on the degree of reproductive isolation among lineages and the historical factors driving divergence [2]. In Europe, glacial dynamics over the last several million years had a major impact on the phylogeography of animal and plant lineages, which underwent repeated cycles of rapid expansions from southern refugia as species recolonized northern latitudes following glacial maxima [3-5]. These range expansions gave rise to a series of suture zones across the continent as lineages that diverged during glacial periods came into secondary contact during interglacials, providing us with unique natural experimental areas in which to study the process of lineage divergence and the evolution of reproductive isolation [5].

One of these regions of secondary contact is the Ibero-Pyrenean suture zone, an important biogeographic region where both inter and intra-specific lineages are known to come into contact in a number of taxa [3-6]. In this area of southwestern Europe, the Pyrenees stand as a major barrier separating central Europe from the Iberian peninsula, one of the major glacial refugia in southern Europe, and also a geologically and ecologically complex area that has suffered itself severe climatic changes, giving rise to a complex phylogeographic pattern that consists of “refugia within refugia” [7]. Here we examine the phylogeography and patterns of gene flow among lineages of the common lizard *Zootoca vivipara* (= *Lacerta vivipara*) in the Ibero-Pyrenean region. The species has a broad Eurasian distribution composed largely of viviparous lineages, yet individuals in this region belong to an oviparous lineage isolated from the nearest viviparous populations in the French Massif Central by a gap of unsuitable habitat [8]. Recent studies of *Z. vivipara* in this region have documented a sharp contact zone between two divergent maternal lineages corresponding generally to the French and Spanish sides of the Pyrenees, and showing consistent results with mtDNA 16S and cytochrome *b* gene sequences [8,9] and a maternally inherited marker on the female W chromosome [10]. However, because of limited regional sampling in previous phylogeographic studies, and the lack of biparentally inherited markers examined in this system to date, our understanding of region-wide genetic structure, patterns of introgression among lineages and the relative importance of the Pyrenees and other mountain ranges as barriers to gene flow remain general and tentative.

Inferring the demographic and evolutionary history of lineages at various spatial and temporal scales often requires the use of molecular markers from genomic regions with different modes of inheritance and rates of evolution [11]. Mitochondrial DNA sequence markers have proven useful for studying intraspecific genetic variation because of their relatively high mutation rate, small effective population size, short coalescence times, and matrilineal mode of inheritance [1,12,13]. However, mt-DNA genomes are susceptible to the effect of historical factors such as population expansions [3,14], introgressive hybridization [15,16], incomplete lineage sorting [17] and even selection [18,19], all of which can effectively mask real patterns of gene flow among populations and lineages, potentially confounding inferences of evolutionary and demographic events [20]. Complementing mtDNA datasets with biparentally inherited nuclear DNA (nDNA) marker data is often essential to obtain a more complete picture of lineage history [11,21].

Here we undertake a multilocus phylogeographic survey across the region using both mtDNA sequences and genome-wide AFLP loci and an improved geographic sampling in order to document the presence and distribution of major genetic lineages and test the role of the Pyrenees and the Cantabrian Mountains in driving lineage divergence in *Z. vivipara*. We also use this large-scale phylogeographic context to assess patterns of introgression and test hypotheses related to the history of contact zones.

## Results

### Mitochondrial DNA structure and diversity

Sequencing of 200 individuals of *Z. vivipara* from 48 localities (Table 1) revealed 14 cyt-*b* and 29 ND2 haplotypes, for a total of 33 haplotypes for the concatenated dataset (1,370 bp).

**Table 1:** Sampling localities, sample sizes and mtDNA haplotype frequencies

**Locality**	**Loc. code**	**Latitude**	**Longitude**	**n**	**mtDNA haplotypes**
**Spain**					
Xistral	XIS	43,46678	−7,53726	9	XJ(7), X1J(2)
Piornedo	PIO	42,84147	−6,86184	2	MI(2)
Somiedo	SOM	43,00245	−6,22712	5	MI(5)
Cofiñal	COF	43,05485	−5,27876	5	NI(5)
Andara	AND	43,21778	−4,71355	5	LI(4), L1I
Estacas	ESC	43,12082	−3,67084	14	GH(11), GG(3)
Barazar	BAR	43,05901	−2,71591	5	GH(1), IF(2), JH(1), KH(1)
Irun	IRU	43,33072	−1,80361	13	FH(1), GH(1), HH(11)
Lizaso	LIZ	42,96035	−1,67535	10	GH(10)
Ibañeta	IBA	43,02089	1,31989	10	GH(5), AA(2), BC(1), CC(2)
Guarrinza	GUA	42,84706	−0,65712	11	AA(6), AB(3), EA(2)
Somport	SOP	42,79871	−0,53144	12	AA(11), DA(1)
Turbera	TUR	42,79968	−0,41439	13	AA(13)
Zuriza	ZUR	42,85636	−0,79271	5	AA(1), AB(4)
Beret	BET	42,71769	0,95585	6	PM(5), QM(1)
**France**					
Ayguebere	AYG	42,89613	−0,44424	7	AA(5), SL(2)
Aran 1	AR1	43,05057	−0,53855	1	TL(1)
Aran 2	AR2	43,04700	−0,52374	1	TK(1)
Col d’Aubisque	AUB	42,97254	−0,34547	1	TL(1)
Benou	BEN	43,06322	−0,45656	1	TK(1)
Col de Bergout	BER	42,97693	−0,56600	1	TK(1)
Plateau de Bious	BIO	42,86300	−0,45400	1	DA(1)
Gave du Baralet	BLT	42,87020	−0,58232	1	AA(1)
Col de Bouesou	BOE	43,00468	−0,66954	1	AA(1)
Bosdapous	BOS	43,05911	−0,61905	1	AE(1)
Brousset	BRO	42,85126	−0,38934	9	AA(9)
Soussouéou bas	SOU	42,90145	−0,36631	10	AA(4), TL(1), WL(5)
Biscau	BSC	42,89844	−0,45962	1	AA(1)
Barescou	ES1	43,07716	−0,59873	1	TK(1)
Pacq	ES2	43,07869	−0,61700	1	AA(1)
Sarrances-Penecq	ES4	43,06390	−0,60130	10	AA(5), TK(5)
Estaing-Lac	EST	42,90980	−0,22923	1	ZN(1)
Lac de Fabrege	FBR	42,88034	−0,40263	1	AA(1)
Gabas-piet	GAB	42,89696	−0,42331	9	AA(5), SL(1), WL(3)
Gloutaret	GLO	43,02429	−0,47512	1	S1L(1)
Col d’Inharpu	INH	43,09557	−1,03068	1	AD(1)
Iraty	IRA	43,04206	−1,07265	1	AA(1)
Grange Ire	IRE	42,99365	−0,52301	1	SL(1)
Laberouat	LAB	42,94946	−0,66441	1	AA(1)
Madeleine	MAD	43,14639	−0,83911	1	AA(1)
Col de Marie-Blanque	MB1	43,07075	−0,50787	1	TL(1)
Moura de Montrol	MOU	43,50600	−1,29400	3	RL(2), T1L(1)
Col de l’Ours	OUR	42,89791	−0,38302	1	WL(1)
Col des Palomieres	PAL	43,05500	0,15800	1	YL(1)
Peyrenere	PEY	42,80262	−0,54698	1	AA(1)
Pinet-Belesta	PIN	42,70700	2,60100	1	UM(1)
St. Raphael	RAP	44,91600	−0,72700	1	TL(1)
Soussouéou haut	SOH	42,88225	−0,33895	1	WL(1)

The first letter of each haplotype designation corresponds to the ND2 haplotype, and the second one to the cyt-*b* haplotype. For the GenBank accession associated with each haplotype see Additional file 1: Table S1.

**Figure 1:**
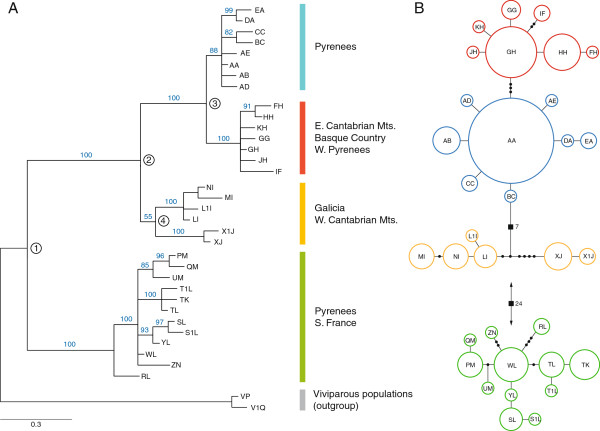
**Phylogenetic relationships among *Zootoca vivipara *mtDNA haplotypes. (A)** Bayesian phylogram based on the concatenated dataset (ND2 + cyt-*b*, 1370 bp). Node support values correspond to posterior Bayesian. **(B)** Statistical parsimony network of mtDNA haplotypes, where each circle represents a haplotype and its size is proportional to the haplotype’s frequency. Branches represent one nucleotide change, black dots indicate additional changes or missing haplotypes, and black squares indicate the number of changes when greater than 4 (branch lengths not drawn to scale). Colours correspond to clades in **(A)**.

**Figure 2:**
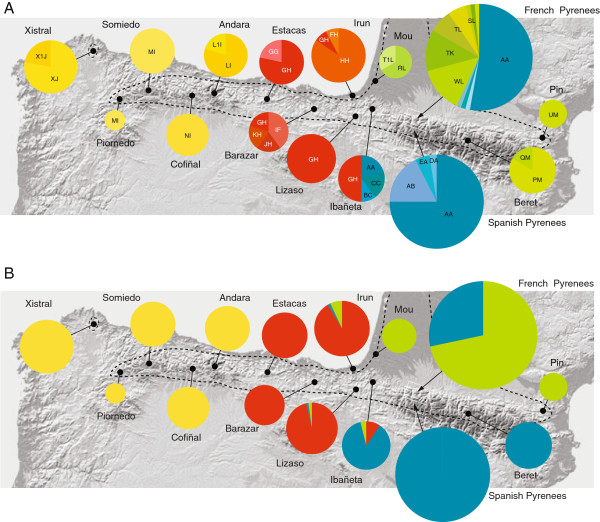
**Geographic pattern of genetic variation in *Zootoca vivipara*. (A)** Frequency of mtDNA haplotypes (concatenated ND2 and cyt-*b* sequences) per locality. The size of each pie graph is proportional to the sample size at each locality (see Table 1). Colours correspond to the major clades from the phylogenetic tree on Figure 1. In the French Pyrenees population six haplotypes with a frequency of one are unlabelled: S1L, YL and ZN (green tones) and AD, AE and DA (blue tones). **(B)** Genetic variation in genome-wide AFLP markers. Each pie represents the posterior probabilities of assignment to four main genetic clusters (K = 4) obtained from Structure 3.1, averaged across individuals at each locality. The size of each pie graph is proportional to sample size. Black dots represent sampling localities, and white frames represent areas including several localities, shown in detail on Figure 3. The dashed contour line represents the approximate distribution range of the species in this region.

**Figure 3:**
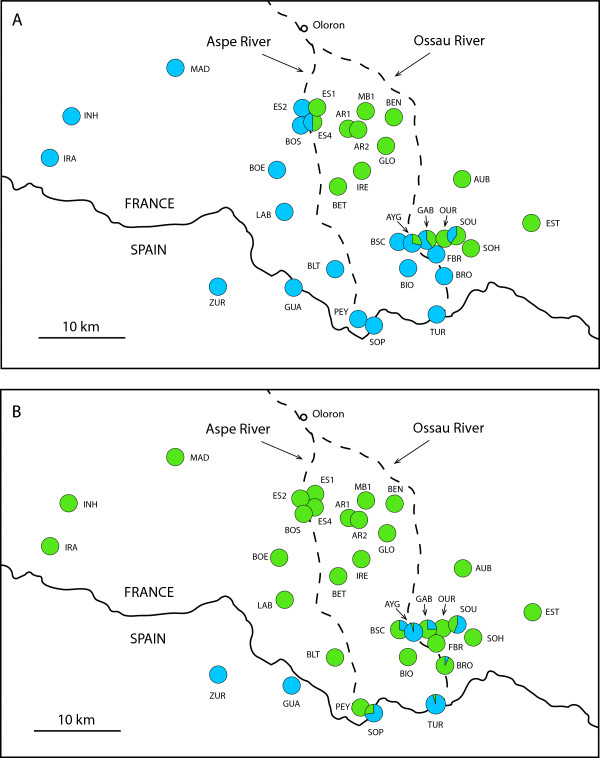
**Genetic patterns at a *Zootoca vivipara *contact zone in the central Pyrenees as reflected by (A) mtDNA sequence data and (B) genome-wide AFLP data.** Colours correspond to lineages in Figure 2.

Phylogenetic analysis revealed two major clades corresponding generally to southern France and northern Spain, respectively (Figures 1 &2A). The Spanish clade is further divided into three major subclades, including a NW Spain clade (Western Cantabrian Mountains), a north-central Spain clade (Eastern Cantabrian Mountains and Basque Country) and a NE Spain clade (Pyrenees) (Figures 1 &2A). Lizards carrying haplotypes from the latter clade were also found on the French slope of the Pyrenees, where they were found sympatrically with the divergent French clade haplotypes (Figures 2A and 3A). Percent divergence in corrected distances between the two major clades was 2%, and average divergence among northern Spain clades was 0.6% (Table 2). Dating of the main clades using Bayesian inference in BEAST, indicate that the main France-Spain split (node 1 on Figure 1A) took place about 0.935 My ago (95% HPD: 0.396-1.591), whereas the divergence among clades in northern Spain ranges from 0,408 My (95% HPD: 0.166-0.713) for node 2, to 0.169 My (95% HPD: 0.056-0.307) for node 3 (Figure 1A).

**Table 2:** **Genetic divergence among *Zootoca vivipara *mtDNA lineages**

	**Yellow**	**Red**	**Blue**	**Green**
NW Spain (yellow)	*0.33*	0.99	0.90	1.98
NC Spain (red)	0.79	*0.06*	0.35	2.31
NE Spain (blue)	0.72	0.30	*0.03*	2.18
S. France (green)	1.71	2.17	2.05	*0.22*

Shown are percent pairwise differences between lineages (above diagonal), percent pairwise differences within lineages (along diagonal), and percent pairwise differences between lineages corrected for intrapopulation polymorphism (below diagonal). Based on 1,370 bp of mtDNA sequence. Colours mentioned in the first column correspond to those on Figures 1 and 2.

Patterns of genetic diversity revealed that the NE Spain clade (blue in Figures 1 &2) showed lower haplotypic and nucleotide diversity values than the rest (Table 3). The prominent “starlike” phylogenetic pattern in this clade, with a single high-frequency haplotype (“AA”) and various lower-frequency, closely-related haplotypes, is suggestive of a recent population expansion [22]. This was corroborated by results from Fu’s test of population expansion, which revealed significant values of F_s_, consistent with a recent burst of population growth (Table 3). The pattern of haplotype frequency and distribution of the NE Spain (blue) clade reflects higher haplotype diversity on the Spanish side of the Pyrenees than on the French side, where the relative frequency of haplotype “AA” is more pronounced. This pattern, together with the evidence for a recent population expansion in this lineage, suggests that the presence of Spanish haplotypes on the French side is the result of a trans-Pyrenean colonization of the French side. We estimated time since the population expansion from the distribution of pairwise differences among blue-clade haplotypes, which yielded a value of τ = 3.00 (95% CI: 0.00-3.83). Applying a mutation rate of 0.01 s/s/Myr per lineage, this value corresponds to a time since the expansion of 54,744 years, with confidence intervals between the present and 69,890 years ago.

**Table 3:** **Sample sizes and diversity indices for the major mtDNA lineages of *Zootoca vivipara***

**Lineage**	**n**	**haps**	***h***	***π***	**Fs**
NW Spain (yellow)	26	6	0.816 ± 0.035	0.0033 ± 0.0019	3.04
NC Spain (red)	47	7	0.596 ± 0.067	0.0007 ± 0.0005	−2.49
NE Spain (blue)	86	8	0.333 ± 0.065	0.0003 ± 0.0003	−5.67**
S. France (green)	41	12	0.868 ± 0.028	0.0022 ± 0.0013	−2.12

Colours in the first column correspond to those on Figures 1 and 2A. For each lineage we provide the sample size (n), the number of haplotypes (haps), haplotype diversity (*h*), nucleotide diversity (*π*), and the Fs value from Fu’s test of neutrality to test for a population expansion.

** P < 0.01.

### AFLP structure and diversity

**Figure 4:**
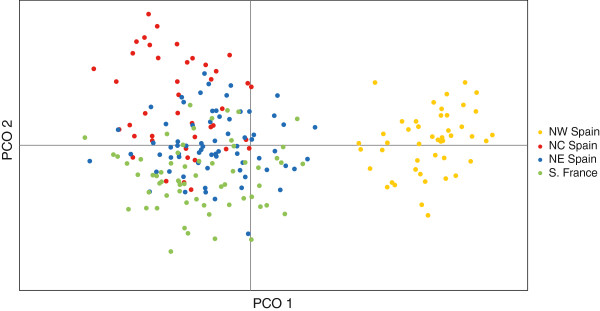
**Principal coordinates analysis (PCoA) of genetic variation in *Zootoca vivipara *based on 34 AFLP loci.** The variance explained by PCO1 and PCO2 is 31% and 19%, respectively. The colours of the dots correspond to the lineages identified by the Bayesian assignment test illustrated in Figure 2B.

**Figure 5:**
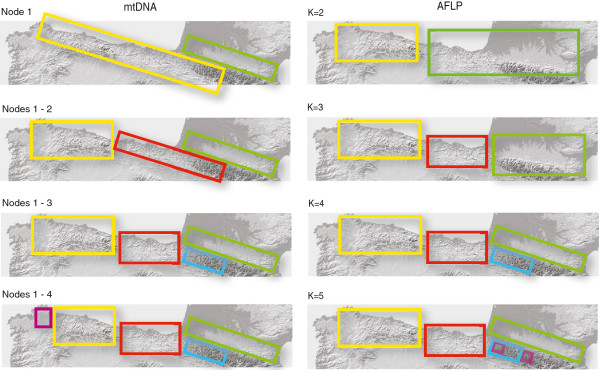
**Summary of patterns of genetic structure recovered by mtDNA markers (left) and genome-wide AFLP markers (right).** MtDNA structure corresponds to the clades in the Bayesian phylogenetic tree in Figure 1, and K values in the AFLP plots correspond to those in an analysis using Structure 2.3.

A total of 34 genome-wide amplified fragments were unambiguously scored for 223 individual lizards using 3 pairs of selective primers. Out of the total 34 loci, 53% to 82% were polymorphic depending on the population (Table 4). Analysis of AFLP variation revealed marked geographic structure that shows partial congruence but also important differences with that found using mtDNA sequence data. A principal coordinate analysis (PCoA) of AFLP variation revealed a major break and the remaining populations to the east (Figures 4 &5), in contrast to the mtDNA pattern which showed a major division separating French and Spanish populations (Figures 2 &5). The Bayesian assignment analysis using the program Structure 3.2 was consistent with the PCoA for K = 2, separating the west-Cantabrian populations from the rest, K = 3 grouped individuals into NW Spain, Basque Country and Pyrenees, K = 4 separated NW Spain, Basque Country, French Pyrenees and Spanish Pyrenees, and K = 5 revealed small additional groups to those in K = 4 (Figure 5). The Evanno method for obtaining the “optimal” K value gave the highest probability to K = 2, with higher values being less likely (ΔK values: 222.82 for K = 2, 72.14 for K = 3, 50.72 for K = 4, and 22.72 for K = 5, Additional file 1: Figure S1A). In contrast, a plot of mean values of the estimated Ln probability of K suggests that K = 4 could correspond to the most realistic level of structure (Additional file 1: Figure S1B), as it marks the start of the flattening of the curve. Determining the optimal K is not always straightforward and consideration of both probability scores and biological information is recommended when assessing the “true” number of populations [23]. Interestingly, K = 4 showed a remarkable geographic correspondence with the four main clades in the mtDNA phylogenetic tree (Figures 2 and 5), strongly suggesting that this K value for AFLP data is biologically meaningful. In any case, the geographic congruence between K = 4 AFLP clusters and the mtDNA phylogenetic tree provides a unique opportunity to compare patterns of divergence between the two types of markers and examine the behaviour of each one at *Z. vivipara* contact zones in the region.

**Table 4:** **Genetic diversity of AFLP loci in four major *Zootoca vivipara *genetic clusters**

**Group**	**n**	**No.**	**%**	***H***_***e***_	**SE (*H***_***e***_**)**
		**loci**	**polymorphic loci**		
NW Spain (yellow)	49	34	52.9	0.193	0.028
NC Spain (red)	42	34	82.4	0.310	0.030
NE Spain (blue)	70	34	82.4	0.317	0.028
S. France (green)	62	34	76.5	0.276	0.029

Colours in the first column correspond to those on Figure 2B.

With respect to genetic differentiation between AFLP genetic clusters (using K = 4), Φ_PT_ between NW Spain (yellow lineage in Figure 2) and all remaining populations was 0.30 (P < 0.001), with Φ_PT_ values between the yellow cluster and other eastern clusters ranging from 0.32 to 0.41 (Table 5).

**Table 5:** **Genetic differentiation among *Zootoca vivipara *lineages based on AFLP data**

NW Spain (yellow)	0	0.183	0.136	0.163
NC Spain (red)	0.405	0	0.054	0.069
NE Spain (blue)	0.321	0.160	0	0.031
S. France (green)	0.367	0.187	0.106	0

Below diagonal: Pairwise values of Φ_PT_ (a statistic analogous to F_ST_). All values are significant at the 0.01 level based on 10,000 permutations. Above diagonal: Nei’s genetic distances (after Lynch and Milligan 1994). Colours in the first column correspond to those on Figure 2B.

None of the 34 loci showed significant departures from neutrality in the BayScan 2.0 analysis across all comparisons, although one of them (locus “15c194”) was identified as an outlier in the K = 2 comparison, and another one (locus “19c84”) in the K = 3 and K = 4 comparisons (Additional file 1: Figure S2). Excluding these two loci from the dataset yielded results for the Structure analysis and the PCoA analysis in Genalex that were indistinguishable from those using all loci. This is due to the fact that most markers in these comparisons had F_ST_ values above 0.20 (mean across-loci F_ST-K2_ = 0.27, SD = 0.01; Mean F_ST-K3_ = 0.25, SD = 0.01; Mean F_ST-K4_ = 0.23, SD = 0.02) and thus most markers contributing to the pattern of genetic structure were selectively neutral.

### Introgression at contact zones

Using the geographic distribution of the main four mtDNA clades and the comparable AFLP-defined genetic units corresponding to K = 4, we detected localities with mixed haplotypes (for mtDNA) or assignment probability values (for ALFPs) in two main areas within the region, one corresponding to the contact zone between blue and green lineages across the Pyrenees, and one between blue and red lineages in Navarre, just east of the Basque Country (Figure 2).

**Figure 6:**
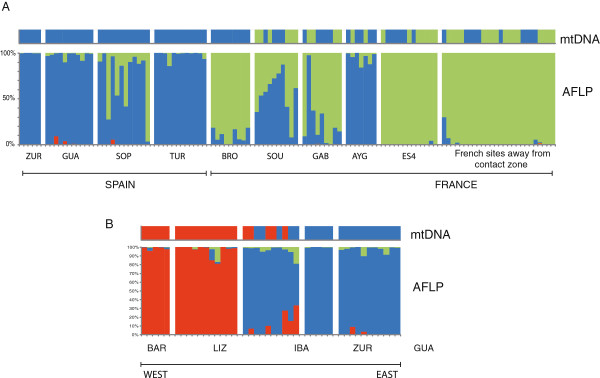
**Introgression patterns in mtDNA and AFLP loci at two contact zones among *Zootoca vivipara *lineages: (A) North–south contact zone between Spain and France; and (B) East–west contact zone between eastern and central Pyrenees.** Each plot is divided into an mtDNA block on top and an AFLP block below. Each vertical bar on these blocks corresponds to a single individual in the different sampling sites (abbreviated on the bottom row). For the mtDNA, colors correspond to clades in Figure 2A, and for the AFLP data each vertical bar corresponds to the percent assignment probability to genetic clusters on Figure 2B.

The contact zone in southern France between divergent blue and green mtDNA lineages, apparent in Figures 2A and 3A and previously described in detail by Heulin et al. [24] and references therein, is shifted to the south according to genome-wide AFLP data. Populations containing genotypes assigned to blue and green genetic clusters are restricted to a limited area within a few kilometres of the Spanish border (upper Ossau River), whereas the genotypes of individuals in the remaining area to the north group with the green southern France cluster (Figure 3B). At the AFLP contact zone, the degree of nuclear introgression varies per population (Figure 6A), with most individuals in some localities showing high to complete assignment probability to either the blue or green clusters (AYG and BRO, respectively), whereas in others (SOU, GAB and SOP) individuals show a range of assignment probabilities suggesting a hybrid origin. The ES4 population and other sites located further away from the border show complete assignment probability to the southern France (green) cluster.

At a second mtDNA contact zone detected between the red and blue clades at the Ibañeta site in the Spanish side of the western Pyrenees (Figure 2A), the AFLP data revealed that all individuals had a higher probability of assignment to the blue cluster (Figure 2B). A more detailed examination of the 10 individuals genotyped from this site shows that six of them had >90% probability of assignment to the blue cluster and less than 10% to the red cluster, and the remaining four individuals had 86-48% probability of assignment to the blue cluster and 10-33% to the red cluster (Figure 6B).

A final discrepancy between the two datasets pertains to the assignment of the Beret site (BER), near the border in the central Pyrenees, where the six individuals sampled carry southern France mtDNA haplotypes (green clade), yet their AFLP profiles cluster with the NE Spain group (blue cluster) (Figure 2), a situation similar to that of many southern France individuals.

## Discussion

### Phylogeography and mtDNA lineage history

Our mtDNA dataset revealed four major clades and a remarkable degree of geographic structuring among *Zootoca vivipara* lineages. The high variation found in the ND2 gene relative to cyt-*b*, together with a more thorough geographic sampling allowed us to identify several distinct and geographically segregated clades in northern Spain, which help explain their historical interactions with the divergent clade found in southern France and clarify the phylogeographic history of the region.

In line with previous phylogeographic studies on the species, our study shows that populations of *Z. vivipara* at the Ibero-Pyrenean zone do not show an actual “suture zone” between Iberian and northern European lineages [8,24], in sharp contrast to patterns found in other species, like the grasshopper *Chorthippus parallelus*[25] or the warbler *Phylloscopus collybita*[26]. We also find no evidence of Iberian clades expanding northward to colonize northern European latitudes, as documented for toads [27], shrews [28], hedgehogs [29], or bears [30]. Instead, the oviparous clades of *Z. vivipara* on opposite sides of the Pyrenees form a monophyletic clade with respect to all other European lineages, indicating a relatively long time in isolation in this region. However, our mtDNA data do support a major role for the Pyrenees in isolating populations, and the two main divergent lineages (depicted by node “1” in Figure 1A) appear to have diverged on either side of this major barrier within the last one million years, since around the mid Pleistocene. The Spanish clade further differentiated within the last 500 K years into at least three subclades corresponding to separate mountainous regions, namely the western and eastern sections of the Cantabrian Mountains, and the south-facing slope of the Pyrenees. Further structure is also indicated by the small clade in the western-most end of the range, formed by two haplotypes (XJ and X1J) sampled in the Serra do Xistral, Galicia, although further sampling will be necessary to confirm this pattern.

Overall, the general pattern of allopatric differentiation in isolated highlands is consistent with the species ecology, as in the southern part of its distribution the common lizard is found mainly in humid bogs and associated habitats [31,32], which in Spain are often found in montane areas. Other lizards associated with humid areas, such as members of the genus *Iberolacerta,* show a deep phylogenetic break between the NW Spain form (*I. monticola*) and the Pyrenean taxa (*I. bonnali*, *I. aranica* and *I. aurelioi*), suggesting similar patterns of isolation as in *Z. vivipara* albeit at longer time scales [33,34]. In contrast, species that do not specialize on humid habitats have shown a contrasting lack of structure across similar regions. For instance, populations of *Lacerta schreiberi* along northern Spain are genetically uniform and expanded from a refuge in central Portugal since the last glacial maximum [35], a pattern also shown by *Podarcis bocagei*[36] and the salamander *Chioglossa lusitanica*[37].

The pattern of distinct *Z. vivipara* lineages associated with different mountain areas along the northern Iberian peninsula is consistent with a pattern of “refugia-within-refugia” documented for a number of other organisms [7,38,39], and is consistent with a climatically dynamic history which combined with a complex topography has given rise to allopatrically differentiated populations and lineages. In this context, *Z. vivipara* lineages are younger than those reported in other taxa, such as *Psammodromus hispanicus*[39], *Alytes obstetricans*[40], or *Lissotriton boscai*[41], and more similar in age to those in *Alytes cisternasii*[42].

### AFLP structure: contrasting patterns with mtDNA

The pattern of spatial variation in genome-wide AFLP markers revealed both important similarities and differences compared to that of mtDNA sequence. Both datasets revealed the presence of four genetic units that are geographically concordant, yet relative divergence among groups differs in both datasets. AFLP variation forms two main genetic groups that separate NW Spain from the rest, while the two main mtDNA clades separate Spain from France. This difference suggests that at a large geographic scale different neutral and/or selective factors have shaped diversity and structure in both types of markers, yet identifying the specific cause is beyond the scope of our dataset. Potential causes range from neutral factors such as random sorting of loci or chromosomal rearrangements, to the role of natural selection. Even though the AFLP loci we used appear to be largely neutral, these markers are likely to be spread randomly across the nuclear genome, and are therefore more likely to be associated with regions under selection than mtDNA markers. If this were the case, we would expect to find a stronger association between traits under selection and AFLP structure. Available phenotypic data on *Z. vivipara* in the region provides some support for this hypothesis, and analysis of morphological traits has revealed marked phenotypic differentiation of Cantabrian populations with respect to those in the Basque Country and Pyrenees [43]. This pattern is driven largely by the shape of the head-shield scales, a trait with unknown fitness value, although other traits related to coloration known to be important in related lacertids [44,45] are yet to be analysed in *Z. vivipara*. A closer association between traits under selection and nuclear instead of mitochondrial neutral makers has been documented in other studies. For example, in a contact zone between central European newts, Babik et al. [46] document a tighter correlation between nuclear DNA and morphological hybrid indices than between mtDNA and morphology. Also, a recent study in the *Ensatina* salamander complex of North America shows that divergence in neutral nuclear markers is better correlated with reproductive isolation than mtDNA [47]. Additional ecological and phenotypic data will be necessary to properly test the hypothesis that neutral nuclear markers show a better association to adaptive variation than mitochondrial markers.

### Contact zone dynamics and patterns of introgression

Our phylogeographic results reveal the presence of two independent contact zones between lineages: one in southern France across the high Pyrenees (between the blue and green clades in Figure 2) and one in the foothills of the southwestern Pyrenees (between the red and blue clades). An additional contact zone in the Basque lowlands between the red and green clades was not detected here due to limited sampling in that area, but a population with haplotypes from both clades has been reported by Heulin et al. (2011) at Moura de Montrol sites. Patterns of introgression in this area have been studied in detail among lineages of *Corthippus* grasshoppers [48] and *Phylloscopus* birds [26,49], both forming suture zones among old lineages. Additional spatial sampling will be necessary to properly compare these suture zones to the secondary contact dynamics among the relatively young lineages of *Z. vivipara*. Another potential contact zone not detected in the present study corresponds to that found in north-central Spain, between the yellow and red lineages, and further sampling would be necessary to confirm its existence.

Relatively good sampling in the central Pyrenees allows us to infer the demographic and introgression history of lineages there. Different lines of evidence (phylogeny, haplotype frequencies and expansion test) support the hypothesis that the presence of NE Spanish (blue) haplotypes in southern France is due to a recent population expansion from the Spanish side of the Pyrenees, which led to the colonization of southern France across the high Pyrenees. The marked predominance of haplotype “AA” there suggests that this incursion took place recently, probably during or since the last glacial maximum, as indicated by time estimates from the mismatch distribution. The relative geographic congruence among mtDNA and AFLP genetic groups allows us to compare patterns of introgression of maternal and bi-parentally inherited loci. Genome-wide AFLP data revealed limited introgression of nDNA into France, with individuals of mixed origin being restricted to the upper Ossau river drainage and the Spain-France border, suggesting that the contact zone among lineages is located within 10 or 15 km of the border. In contrast, Spanish mtDNA haplotypes are found at least tens of kilometres further than nDNA. Interestingly, it appears that Spanish haplotypes expanded into France (mostly towards the W and NW), they were then trapped at river valleys towards the east (i.e. lower Aspe river and upper Ossau river valleys), giving rise to a sharp contact zone there. This apparent barrier to gene flow in mtDNA but not nDNA at river valleys could be due indirectly to Haldane’s rule [50], which predicts a lower fitness for hybrids of the heterogametic sex, females in the case of *Z. vivipara*. A more direct role of selection through differential survivorship of alternative haplogroups has been previously suggested by Heulin et al. (2011), although recent evidence from the same study area in Gabas-piet (Heulin, unpublished data) indicates that further replication across years will be necessary to obtain conclusive evidence. Several studies in diverse organisms have shown that mtDNA can introgress further than nDNA [20,46,51-54], and spread relatively quickly. An alternative explanation for the shifted positions of the mtDNA and nDNA clines is that following the initial expansion, the contact zone has moved back south, leaving behind Spanish mtDNA haplotypes in its wake [55,56]. Distinguishing among these alternative scenarios will require further geographic and molecular sampling, including co-dominant nuclear markers.

Additional fine-scale sampling at contact zones among lineages will also be needed to understand introgression dynamics at other areas, such as the Beret site, where all individuals carry French mtDNA and Spanish nDNA, or the contact zone in the western Pyrenees between the blue and red clades detected at the Ibañeta site, where individuals show blue central-Pyrenean nDNA, yet half of them carry red mtDNA haplotypes. Patterns at these individual sites are similar to those found at some sites in southern France, and a more thorough geographic context provided by finer-scale sampling will be needed to help determine whether they belong to active contact zones with ongoing introgression or instead represent populations left in the genetic wake of a contact zone that shifted away.

## Conclusions

Our results show that lineages of *Z. vivipara* in northern Iberia and the Ibero-Pyrenean suture zone show marked levels of differentiation that is congruent with major topographical features in the region. Our data reveal that mountain ranges do not necessarily represent impassable barriers for this species, as demonstrated by a recent high-elevation colonization event across the Pyrenees that has led to secondary contact and partial introgression among divergent lineages. In contrast, we find indirect evidence of contact zones in lowland areas, in the absence of obvious barriers to dispersal. Our results underscore the importance of multilocus datasets in reconstructing the evolutionary history of independent intraspecific lineages [57], and understanding the dynamics of introgression upon secondary contact. The degree of mixing among *Z. vivipara* lineages in secondary contact is similar to that found among different lizard species [58], suggesting that *Z. vivipara* lineages in the region may in fact represent separate species. Further research using both molecular and phenotypic data will help determine the extent to which reproductive isolation is underway despite the presence of gene flow [59]. Suture zones are important areas for the study of lineage formation and speciation, as they provide a geographic context for across-taxa comparisons. However, our results on *Z. vivipara* show that even major biogeographic barriers can yield markedly different phylogeographic patterns in different vertebrate and invertebrate taxa depending on their demographic histories and ecological traits. Combining intraspecific studies of genetic variation at the lineage level with ecological data will be essential to better understand phylogeographic differences among taxa and advance our understanding of diversification mechanisms.

## Methods

### Field sampling

Specimens of *Z. vivipara* were captured at 49 localities across the species range in Northern Spain and Southern France (Table 1). The tail tip of each individual captured was clipped and stored in 95% ethanol at −20°C. Individuals were weighed, measured, photographed, and then released at the site of capture. The capture and handling of lizards complied with national and international ethical guidelines and was conducted under the scientific collecting permits issued by Xunta de Galicia, Junta de Castilla y León, Gobierno del Principado de Asturias, Gobierno de Navarra, Gobierno de Aragón and Generalitat de Catalunya in Spain, and Parc National des Pyrénées in France.

### MtDNA sequencing and analysis

Genomic DNA was extracted from tail clippings using a Qiagen™ DNeasy Kit and following the manufacturer’s protocol. The following regions of the mitochondrial DNA were amplified: a 427 bp fragment of the cytochrome *b* gene (including 21 bp from the adjacent Glu-tRNA) using primers MVZ04 and MVZ05 [60]; and 964 bp of the NADH dehydrogenase subunit 2 gene (ND2) using primers MetF6 and AsnR2 [61]. Detailed PCR amplification conditions and sequencing details are available in the Additional file 1.

Sequences were automatically aligned using Sequencher 4.1. (GeneCodes, Ann Arbor, Michigan) and variable sites were checked visually for accuracy. Coding gene sequences were unambiguously translated into their amino acid sequence and no double peaks were observed in the chromatographs, suggesting sequences were of mitochondrial origin and not nuclear copies. For most analysis, the two gene fragments (cyt-*b* and ND2) were concatenated into a single sequence of 1370 bp. Haplotype and nucleotide diversity indices were calculated in DnaSP v5[62].

We constructed a haplotype network using a maximum parsimony algorithm as implemented in the program TCS 1.21 [63], setting minimum branch probability at 95%. We used jModelTest[64] to determine the model of sequence evolution for each marker (HKY + G for ND2 and HKY + I for Cyt-*b*) and obtained an optimal partitioning scheme for the dataset (by gene and by codon position) using the sotware package PartitionFinder [65]. We then constructed a phylogeny of mtDNA haplotypes using Bayesian analysis with MrBayes 3.2.2 (http://mrbayes.csit.fsu.edu/) [66], and ran two simultaneous parallel runs of four Markov chains each (3 heated and one cold) for 4 million generations and sampled every 100 generations. The first 25% of the trees were excluded as burn-in, and a consensus topology was obtained from the remaining 60,002 samples (30,001 per run). We confirmed MCMC chain convergence using Tracer v1.5 [67], and output parameters such as an average PSRF value of 1.00, and an average value of 0.0035 for the standard deviation of the split frequencies between simultaneous runs.

To estimate divergence times among lineages we used a coalescence approach that uses Bayesian inference and MCMC simulations to generate posterior probability values for divergence times as implemented in the program Beast[68]. We partitioned our dataset by gene and codon position, and ran the analysis with unlinked substitution and clock models for each partition, yet generated a single tree from both. We conducted preliminary runs using an uncorrelated relaxed lognormal clock to account for rate heterogeneity among lineages, but obtained a value of zero for the ucld.stdev parameter, indicating that our data are clock-like. We therefore used the strict clock in the final analysis, as well as a coalescent model of diversification, and a UPGMA starting tree. After optimizing the priors with preliminary runs, we conducted two final runs of 20 million generations, sampled every 2000 steps. Chain convergence and burn-in were determined with the program Tracer v1.5 [67], and all ESS values in the final runs were above 600. As a prior for the mutation rate we used a value of 0.0228 subst./site/Myr (0.0114 s/s/Myr per lineage) for Cyt *b*, a rate estimated from a comprehensive phylogenetic study of several squamate groups [69] that used as time calibration points the known age of the Canary Islands and the opening of the Strait of Gibraltar 5.3 Mya, well known geological events that have been associated with speciation events in several reptile and amphibian species [70,71]. We set a lognormal distribution for the prior with a mean of 0.0114 and a log standard deviation of 0.40, so that values of 0.005 and 0.020 s/s/Myr per lineage corresponded to the 5% and 95% quantiles, respectively, as these values encompass the range of rates used for estimating divergence times in other studies on lizards [33,35,38,72].

We tested for past sudden changes in effective population size using Fu’s test of neutrality [73], which detects departures from neutrality in scenarios characterized by an excess of rare alleles and young mutations in sequences not subjected to recombination. Significant large negative values of *F*_*s*_ (generated with Arlequin 3.5) indicate an excess of recent mutations and reject population stasis (Fu, 1997). To estimate the time elapsed since such a sudden population expansion, we used the parameter τ obtained from the distribution of pairwise differences among Cyt *b* haplotypes, as τ = 2*ut*, where *t* is the time elapsed between initial and current population sizes and *u* = 2*μk*, where *μ* is the mutation rate (0.01 substitutions/site/My per lineage) and *k* is the length of the sequence [74]. We estimated the parameter τ using a generalized non-linear least-square approach [75] and computed confidence intervals for an alpha level of 0.05 by a parametric bootstrap method based on 100 replicates as implemented in Arlequin 3.5 [76].

### AFLP profiling and analysis

Amplified fragment length polymorphism (AFLP) profiles were generated using the laboratory protocol described in Milá et al. [77], which was modified slightly from Vos *et al.*[78]. In brief, whole genomic DNA was digested with restriction enzymes *Eco*RI and *Mse*I (*Tru9*) and fragments were ligated to oligonucleotide adapters with T4 DNA ligase. A random sub-sample of fragments was obtained through a pre-selective amplification reaction using primers E_T_ and M_C_, followed by three selective amplifications using primer pairs E_TAG_/M_CGA_, E_TAG_/M_CGT_, and E_TAG_/M_CTA_, with the E primer fluorescently labelled. Ten pairs of selective amplification primers were tested, but only those producing sufficient loci and repeatable and unambiguously scorable profiles were selected for the analysis. Selectively amplified fragments were run in an ABI 3730XL genetic analyser with a LIZ500 size standard. Peaks were visualized using Genemapper 3.7 and scored manually by a single observer (BM). Only unambiguously scorable loci and individuals were included in the analysis and peaks found in less than 2% of individuals were excluded. Methodological error rate was assessed by running a subset of 10 individuals twice from the pre-selective amplification step. The average per-locus genotyping error rate for the AFLP loci selected, measured as recommended by Bonin et al. [79], was low at 1.2%.

To determine whether AFLP markers were selectively neutral, we conducted an outlier analysis using the program BayeScan 2.0, which uses differences in allele frequencies between populations and a reversible-jump MCMC algorithm to estimate the locus-specific posterior probability for a model including selection vs. a neutral model [80]. We used 10,000 iterations, a thinning interval of 100, 40 pilot runs, an additional burn-in of 5,000, and a value of 10 for the prior odds for the neutral model [80].

We estimated allelic frequencies using Zhivotovsky’s [81] Bayesian method with uniform prior distributions and assuming Hardy-Weinberg genotypic proportions. Genetic diversity (*H*_*e*_) was based on allele frequencies calculated using the method by Lynch and Milligan [82] as implemented in the program Aflp-surv v. 1.0 [83]. A matrix of pairwise population *F*_*ST*_ values using the *F*_*ST*_ analogue Φ_PT_ was calculated with Genalex 6.0 [84]. Φ_PT_ is calculated as *V*_AP_/(*V*_AP_ + *V*_WP_) where *V*_AP_ is the variance among populations and *V*_WP_ is the variance within populations. Probability values of pairwise Φ_PT_ were based on 10,000 permutations.

To assess genetic structure among samples we conducted a principal coordinate analysis (PCoA; Orloci [85]) on a genetic distance matrix generated from the binary presence-absence matrix as implemented in Genalex 6.0. [84].We further examined patterns of population structure using the Bayesian assignment probability test in the program Structure 3.1 [86]. This program uses a Bayesian approach to generate posterior probabilities of assignment of individuals to each of a given number of groups (K). As recommended for dominant markers, we applied a model of no admixture with correlated allele frequencies [23], and used the *Locprior* setting, which incorporates sampling locality as a prior without affecting the value of the optimal K [87]. We run five different chains of 1,000,000 steps for each value of K after a burn-in of 100,000 steps. The optimal value of K was estimated by examining the mean Ln likelihood values for different K values from 1 to 10, and by following the method by Evanno et al. [88] as implemented in Structure Harvester[89].

## Availability of supporting data

Sequences used in this study have been deposited in GenBank under accessions for ND2 ( KF593862-KF593891) and Cyt *b* (KF593892-KF593905 and KF593907-KF593908) haplotypes.

## Competing interests

The authors declare that they have no competing interests.

## Authors’ contributions

BM, YSG, BH and PF designed the study; all authors carried out the field sampling; BM and YSG generated the molecular data; BM conducted all genetic analyses; BM wrote the manuscript with contributions from all authors, who read and approved the final manuscript.

## Supplementary Material

Additional file 1: Table S1 GenBank accessions for each *Zootoca vivipara* mtDNA haplotype used in the study. **Figure S1.** Plots generated by the software Structure Harvester to determine the optimal K in the program Structure 3.1. (A) Delta K values from the method by Evanno et al. (2005). (B) Mean values of the estimated Ln probability of different K values. **Figure S2.** Results from an outlier analysis to detect loci under selection among 34 AFLP loci using the program BayeScan 2.0. Plots show Fst vs. posterior odds for selection for each locus in three population comparisons: (A) K = 2, (B) K = 3, and (C) K = 4. The vertical line in each plot indicates the threshold leading to a false discovery rate (FDR) of no more than 5%. Loci to the right of the line deviate from neutrality for the comparison shown. The loci represented by the dots sitting on the vertical lines are locus “15c194” in (A) and locus “19c84” in (B) and (C). Click here for file

## References

[B1] Avise JC Phylogeography: the history and formation of species 2000 Cambridge, Massachusetts: Harvard University Press

[B2] Barton N Hewitt G Analysis of hybrid zones Ann Rev Ecol Syst 1985 16 113 148 10.1146/annurev.es.16.110185.000553

[B3] Hewitt GM Genetic consequeneces of climatic oscillations in the Quaternary Phil Trans R Soc Lond B 2004 359 183 195 10.1098/rstb.2003.1388 15101575 PMC1693318

[B4] Hewitt GM The genetic legacy of the Quaternary ice ages Nature (London) 2000 405 907 913 10.1038/35016000 10879524

[B5] Taberlet P Fumagalli L Wust-Saucy AG Cosson JF Comparative phylogeography and postglacial colonization routes in Europe Mol Ecol 1998 7 453 464 10.1046/j.1365-294x.1998.00289.x 9628000

[B6] Joger U Fritz U Guicking D Kalyabina-Hauf S Nagy ZT Wink M Phylogeography of western Palaearctic reptiles - Spatial and temporal speciation patterns Zool Anz 2007 246 293 313 10.1016/j.jcz.2007.09.002

[B7] Gómez A Lunt DH Weiss S, Ferrand N Refugia within refugia: patterns of phylogeographic concordance in the Iberian Peninsula Phylogeography of Southern European Refugia 2007 Netherlands: Springer 155 188

[B8] Surget-Groba Y Heulin B Guillaume C-P Thorpe RS Kupriyanova L Vogrin N Maslak R Mazzotti S Venczel M Ghira I Intraspecific phylogeography of *Lacerta vivipara* and the evolution of viviparity Mol Phylogenet Evol 2001 18 449 459 10.1006/mpev.2000.0896 11277636

[B9] Heulin B Surget-Groba Y Guiller A Guillaume C-P Deunff J Comparisons of mitochondrial DNA (mtDNA) sequences (16S rRNA gene) between oviparous and viviparous strains of *Lacerta vivipara*: a preliminary study Mol Ecol 1999 8 10 1627 1631 10.1046/j.1365-294x.1999.00746.x 10583826

[B10] Guillaume C-P Heulin B Arrayago MJ Bea A Braña F Refuge areas and suture zones in the Pyrenean and Cantabrian regions: geographic variation of the female MPI sex-linked alleles among oviparous populations of the lizard *Lacerta (Zootoca) vivipara* Ecography 2000 23 3 10 10.1111/j.1600-0587.2000.tb00255.x

[B11] Edwards SV Beerli P Perspective: gene divergence, population divergence, and the variance in coalescence time in phylogeographic studies Evolution 2000 54 1839 1854 11209764 10.1111/j.0014-3820.2000.tb01231.x

[B12] Zink RM Barrowclough GF Mitochondrial DNA under siege in avian phylogeography Mol Ecol 2008 17 2107 2121 10.1111/j.1365-294X.2008.03737.x 18397219

[B13] Moore WS Inferring phylogenies from mtDNA variation: Mitochondrial-gene trees versus nuclear-gene trees Evolution 1995 49 718 726 10.2307/2410325 28565131

[B14] Excoffier L Foll M Petit RJ Genetic consequences of range expansions Ann Rev Ecol Syst 2009 40 481 501 10.1146/annurev.ecolsys.39.110707.173414

[B15] Milá B Toews DPL Smith TB Wayne RK A cryptic contact zone between divergent mitochondrial DNA lineages in southwestern North America supports past introgressive hybridization in the yellow-rumped warbler complex (Aves: *Dendroica coronata*) Biol J Linn Soc 2011 103 696 706 10.1111/j.1095-8312.2011.01661.x

[B16] McGuire JA Linkem CW Koo MS Hutchison DW Lappin AK Orange DI Lemos-Espinal J Riddle BR Jaeger JR Crandall K Mitochondrial introgression and incomplete lineage sorting through space and time: phylogenetics of crotaphytid lizards Evolution 2007 61 2879 2897 10.1111/j.1558-5646.2007.00239.x 17941840

[B17] Funk DJ Omland KE Species-level paraphyly and polyphyly: frequency, causes, and consequences, with insights from animal mitochondrial DNA Ann Rev Ecol Syst 2003 34 397 423 10.1146/annurev.ecolsys.34.011802.132421

[B18] Bazin E Glémin S Galtier N Population size does not influence mitochondrial genetic diversity in animals Science 2006 28 570 572 16645093 10.1126/science.1122033

[B19] Dowling DK Friberg U Lindell J Evolutionary implications of non-neutral mitochondrial genetic variation Trends Ecol Evol 2008 23 546 554 10.1016/j.tree.2008.05.011 18722688

[B20] Ballard JWO Whitlock MC The incomplete natural history of mitochondria Mol Ecol 2004 13 729 744 10.1046/j.1365-294X.2003.02063.x 15012752

[B21] Knowles LL Statistical phylogeography Ann Rev Ecol Syst 2009 40 593 612 10.1146/annurev.ecolsys.38.091206.095702

[B22] Slatkin M Hudson RR Pairwise comparisons of mitochondrial DNA sequences in stable and exponentially growing populations Genetics 1991 129 555 562 1743491 10.1093/genetics/129.2.555 PMC1204643

[B23] Pritchard JK Wen W Documentation for Structure Software: Version 2 2004 Available at: http://pritch.bsd.uchicago.edu

[B24] Heulin B Surget-Groba Y Sinervo B Miles D Guiller A Dynamics of haplogroup frequencies and survival rates in a contact zone of two mtDNA lineages of the lizard *Lacerta vivipara* Ecography 2011 34 436 447 10.1111/j.1600-0587.2010.06540.x

[B25] Cooper SJ Ibrahim KM Hewitt G Postglacial expansion and genome subdivision in the European grasshopper *Chorthippus parallelus* Mol Ecol 1995 4 49 60 10.1111/j.1365-294X.1995.tb00191.x 7711954

[B26] Helbig AJ Salomon M Bensch S Seibold I Male-biased gene flow across an avian hybrid zone: evidence from mitochondrial and microsatellite DNA J Evol Biol 2001 14 277 287 10.1046/j.1420-9101.2001.00273.x

[B27] Gonçalves H Martínez-Solano I Ferrand N García-París M Conflicting phylogenetic signal of nuclear vs mitochondrial DNA markers in midwife toads (Anura, Discoglossidae, *Alytes*): deep coalescence or ancestral hybridization? Mol Phylogenet Evol 2007 44 494 500 10.1016/j.ympev.2007.03.001 17433723

[B28] Yannic G Basset P Hausser J A new perspective on the evolutionary history of western European *Sorex araneus* group revealed by paternal and maternal molecular markers Mol Phylogenet Evol 2008 47 237 250 10.1016/j.ympev.2008.01.029 18325791

[B29] Santucci F Emerson BC Hewitt G Mitochondrial DNA phylogeography of European hedgehogs Mol Ecol 1998 7 1163 1172 10.1046/j.1365-294x.1998.00436.x 9734073

[B30] Taberlet P Bouvet J Mitochondrial DNA polymorphism, phylogeography, and conservation genetics of the brown bear *Ursus arctos* in Europe Proc R Soc Lond B Biol Sci 1994 B 255 195 200 10.1098/rspb.1994.0028 8022838

[B31] Pérez-Mellado V Ramos MA Lacerta vivipara Jaquin, 1797 Fauna Ibérica, vol 10 1997 Madrid: Museo Nacional de Ciencias Naturales, CSIC 232 242

[B32] Heulin B Guillaume C-P Vacher JP, Geniez M Le lézard vivipare Les reptiles de la France, Belgique, Luxembourg et Suisse 2010 Paris: Biotope, Publications du Museum National d’Histoire Naturelle 394 401

[B33] Crochet P-A Chaline O Surget-Groba Y Debain C Cheylan M Speciation in mountains: phylogeography and phylogeny of the rock lizards genus *Iberolacerta* (Reptilia: Lacertidae) Mol Phylogenet Evol 2004 30 860 866 10.1016/j.ympev.2003.07.016 15012966

[B34] Mouret V Guillaumet A Cheylan M Pottier G Ferchaud AL Crochet PA The legacy of ice ages in mountain species: post-glacial colonization of mountain tops rather than current range fragmentation determines mitochondrial genetic diversity in an endemic Pyrenean rock lizard J Biogeogr 2011 38 1717 1731 10.1111/j.1365-2699.2011.02514.x

[B35] Paulo OS Dias C Bruford MW Jordan WC Nichols RA The persistence of Pliocene populations through the Pleistocene climatic cycles: evidence from the phylogeography of an Iberian lizard Proc R Soc Lond B 2001 268 1625 1630 10.1098/rspb.2001.1706 PMC1088786 11487410

[B36] Pinho C Harris DJ Ferrand N Contrasting patterns of population subdivision and historical demography in three western Mediterranean lizard species inferred from mitochondrial DNA variation Mol Ecol 2007 16 1191 1205 10.1111/j.1365-294X.2007.03230.x 17391406

[B37] Alexandrino J Froufe E Arntzen JW Ferrand N Genetic dubdivision, glacial refucia and postglacial recolonization in the golden-striped salamander, *Chioglossa lusitanica* (Amphibia: Urodela) Mol Ecol 2000 9 771 781 10.1046/j.1365-294x.2000.00931.x 10849293

[B38] Miraldo A Hewitt GM Paulo OS Emerson BC Phylogeography and demographic history of *Lacerta lepida* in the Iberian Peninsula: multiple refugia, range expansions and secondary contact zones BMC Evol Biol 2011 11 170 10.1186/1471-2148-11-170 21682856 PMC3141430

[B39] Fitze PS González-Jimena V San-José LM San Mauro D Aragón P Suárez T Zardoya R Integrative analyses of speciation and divergence in *Psammodromus hispanicus* (Squamata: Lacertidae) BMC Evol Biol 2011 11 347 10.1186/1471-2148-11-347 22129245 PMC3293786

[B40] Martínez-Solano I Gonçalves HA Arntzen JW García-París M Phylogenetic relationships and biogeography of midwife toads (Discoglossidae: *Alytes*) J Biogeogr 2004 31 603 618 10.1046/j.1365-2699.2003.01033.x

[B41] Martínez-Solano I Teixeira D Buckley D García-París M Mitochondrial DNA phylogeography of *Lissotriton boscai* (Caudata, Salamandridae): evidence for old, multiple refugia in an Iberian endemic Mol Ecol 2006 15 3375 3388 10.1111/j.1365-294X.2006.03013.x 16968276

[B42] Gonçalves H Martínez-Solano I Pereira RJ Carvalho B García-París M Ferrand N High levels of population subdivision in a morphologically conserved Mediterranean toad (*Alytes cisternasii*) result from recent, multiple refugia: evidence from mtDNA, microsatellites and nuclear genealogies Mol Ecol 2009 18 5143 5160 10.1111/j.1365-294X.2009.04426.x 19912538

[B43] Arribas OJ Morphological variability of the Cantabro-Pyrenean populations of Zootoca vivipara (Jaquin, 1787) with description of a new subspecies Herpetozoa 2009 21 123 146

[B44] Stuart-Fox D Godinho R Goüy de Bellocq J Irwin NR Brito JC Moussalli A Široký P Hugall AF Baird SJE Variation in phenotype, parasite load and male competitive ability across a cryptic hybrid zone PLOS One 2009 4 e5677 10.1371/journal.pone.0005677 19479073 PMC2682578

[B45] Nunes VL Miraldo A Beaumont MA Butlin RK Paulo OS Association of Mc1r variants with ecologically relevant phenotypes in the European ocellated lizard, *Lacerta lepida* J Evol Biol 2011 24 2289 2298 10.1111/j.1420-9101.2011.02359.x 21812853

[B46] Babik W Szymura JM Rafiński J Nuclear markers, mitochondrial DNA and male secondary sexual traits variation in a newt hybrid zone (*Triturus vulgaris* x *T. montandoni*) Mol Ecol 2003 12 1913 1930 10.1046/j.1365-294X.2003.01880.x 12803641

[B47] Pereira RJ Monahan WB Wake DB Predictors for reproductive isolation in a ring species complex following genetic and ecological divergence BMC Evol Biol 2011 11 194 10.1186/1471-2148-11-194 21733173 PMC3225234

[B48] Buño I Torroja E López-Fernández C Butlin RK Hewitt GM Gosálvez J A hybrid zone between two subspecies of the grasshopper *Chorthippus parallelus* along the Pyrenees: the west end Heredity 1994 73 625 634 10.1038/hdy.1994.170 7989221

[B49] Salomon M Hemin Y Song variation in the chiffchaffs (*Phylloscopus collybita*) of the western Pyrenees – the contact zone btween the *collybita* and *brehmii* forms Ethology 1992 92 265 282

[B50] Haldane J Sex ratio and unisexual sterility in hybrid animals J Genet 1922 12 101 109 10.1007/BF02983075

[B51] Arntzen JW Wallis GP Restricted gene flow in a moving hybrid zone of the newts *Triturus cristatus* and *T. marmoratus* in western France Evolution 1991 45 805 826 10.2307/2409691 28564049

[B52] Martinsen GD Whitham TG Turek RJ Keim P Hybrid populations selectively filter gene introgression between species Evolution 2001 55 1325 1335 11525457 10.1111/j.0014-3820.2001.tb00655.x

[B53] Rohwer S Bermingham E Wood C Plumage and mitochondrial DNA haplotype variation across a moving hybrid zone Evolution 2001 55 405 422 11308096 10.1111/j.0014-3820.2001.tb01303.x

[B54] Chan KMA Levin SA Leaky prezygotic isolation and porous genomes: rapid introgression of maternally inherited DNA Evolution 2005 59 720 729 15926684

[B55] Krosby M Rohwer S A 2000 km genetic wake yields evidence for northern glacial refugia and hybrid zone movement in a pair of songbirds Proc R Soc B 2009 276 615 621 10.1098/rspb.2008.1310 18986973 PMC2660942

[B56] Sequeira F Alexandrino J Rocha S Arntzen W Ferrand N Genetic exchange across a hybrid zone within the Iberian endemic golden-striped salamander, *Chioglossa lusitanica* Mol Ecol 2005 14 245 254 15643967 10.1111/j.1365-294X.2004.02390.x

[B57] Godinho R Crespo EG Ferrand N The limits of mtDNA phylogeography: complex patterns of population fragmentation, expansion and admixture in the highly structured Iberian lizard *Lacerta schreiberi* are only revealed by the use of nuclear markers Mol Ecol 2008 17 4670 4683 10.1111/j.1365-294X.2008.03929.x 18828782

[B58] Pinho C Kaliontzopoulou A Carretero MA Harris DJ Ferrand N Genetic admixture between the Iberian endemic lizards *Podarcis bocagei* and *Podarcis carbonelli*: evidence for limited natural hybridization and a bimodal hybrid zone J Zool Syst Evol Res 2009 47 368 377 10.1111/j.1439-0469.2009.00532.x

[B59] Mallet J Hybridization as an invasion of the genome Trends Ecol Evol 2005 20 229 237 10.1016/j.tree.2005.02.010 16701374

[B60] Smith MF Patton JL Variation in mitochondrial cytochrome b sequences in natural populations of South American akodontine rodents (Muridae: Sigmodontinae) Mol Biol Evol 1991 8 85 103 2002767 10.1093/oxfordjournals.molbev.a040638

[B61] Macey JR Larson A Ananjeva NB Fang ZL Papenfuss TJ Two novel gene orders and the role of light-strand replication in rearrangement of the vertebrate mitochondrial genome Mol Biol Evol 1997 14 91 104 10.1093/oxfordjournals.molbev.a025706 9000757

[B62] Rozas J Sanchez-DelBarrio JC Messeguer X Rozas R DnaSP, DNA polymorphism analyses by the coalescent and other methods Bioinformatics 2003 19 2496 2497 10.1093/bioinformatics/btg359 14668244

[B63] Clement M Posada D Crandall KA TCS: a computer program to estimate gene genealogies Mol Ecol 2000 9 1657 1659 10.1046/j.1365-294x.2000.01020.x 11050560

[B64] Posada D jModelTest: phylogenetic model averaging Mol Biol Evol 2008 25 1253 1256 10.1093/molbev/msn083 18397919

[B65] Lanfear R Calcott B Ho SYW Guindon S Combined selection of partitioning schemes and substitution models for phylogenetic analyses Mol Biol Evol 2012 29 1695 1701 10.1093/molbev/mss020 22319168

[B66] Huelsenbeck JP Ronquist F Nielsen R Bollback JP Bayesian inference of phylogeny and its impact on evolutionary biology Science 2001 294 2310 2314 10.1126/science.1065889 11743192

[B67] Rambaut D Drummond A MCMC Trace Analysis Package (version 1.4) 2007 Available at: http://tree.bio.ed.ac.uk/software/tracer

[B68] Drummond AJ Suchard MA Xie D Rambaut A Bayesian phylogenetics with BEAUti and the BEAST 1.7 Mol Biol Evol 2012 29 1969 1973 10.1093/molbev/mss075 22367748 PMC3408070

[B69] Carranza S Arnold EN A review of the geckos of the genus *Hemidactylus* (Squamata: Gekkonidae) from Oman based on morphology, mitochondrial and nuclear data, with descriptions of eight new species Zootaxa 2012 3378 1 95

[B70] Carranza S Romano A Arnold EN Sotgiu G Biogeography and evolution of European cave salamanders, *Hydromantes* (Urodela: Plethodontidae), inferred from mtDNA sequences J Biogeogr 2008 35 724 738 10.1111/j.1365-2699.2007.01817.x

[B71] Cox SC Carranza S Brown RP Divergence times and colonization of the Canary Islands by *Gallotia* lizards Mol Phylogenet Evol 2010 56 747 757 10.1016/j.ympev.2010.03.020 20307675

[B72] Carranza S Arnold EN Mateo JA Geniez M Relationships and evolution of the North African geckos, *Geckonia* and *Tarentola* (Reptilia: Gekkonidae), based on mitochondrial and nuclear DNA sequences Mol Phylogenet Evol 2002 23 244 256 10.1016/S1055-7903(02)00024-6 12069554

[B73] Fu YX Statistical neutrality of mutations against population growth, hitchhiking and background selection Genetics 1997 147 915 925 9335623 10.1093/genetics/147.2.915 PMC1208208

[B74] Rogers AR Harpending H Population growth makes waves in the distribution of pairwise genetic differences Mol Biol Evol 1992 9 552 569 1316531 10.1093/oxfordjournals.molbev.a040727

[B75] Schneider S Excoffier L Estimation of demographic parameters from the distribution of pairwise differences when the mutation rates vary among sites: application to human mitochondrial DNA Genetics 1999 152 1079 1089 10388826 10.1093/genetics/152.3.1079 PMC1460660

[B76] Excoffier L Lischer HEL Arlequin suite ver 3.5: a new series of programs to perform population genetics analyses under Linux and Windows Mol Ecol Resour 2010 10 564 567 10.1111/j.1755-0998.2010.02847.x 21565059

[B77] Milá B Carranza S Guillaume O Clobert J Marked genetic structuring and extreme dispersal limitation in the Pyrenean brook newt *Calotriton asper* (Amphibia: Salamandridae) revealed by genome-wide AFLP but not mtDNA Mol Ecol 2010 19 108 120 10.1111/j.1365-294X.2009.04441.x 19943891

[B78] Vos P Hogers R Bleeker M Reijans M van de Lee T Hornes M Frijters A Pot J Peleman J Kuiper M AFLP: a new technique for DNA fingerprinting Nucleic Acids Res 1995 23 4407 4414 10.1093/nar/23.21.4407 7501463 PMC307397

[B79] Bonin A Bellemain E Bronken Eidesen P Pompanon F Brochmann C Taberlet P How to track and assess genotyping errors in population genetics studies Mol Ecol 2004 13 3261 3273 10.1111/j.1365-294X.2004.02346.x 15487987

[B80] Foll M Gaggiotti OE A genome scan method to identify selected loci appropriate for both dominant and codominant markers: a Bayesian perspective Genetics 2008 180 977 993 10.1534/genetics.108.092221 18780740 PMC2567396

[B81] Zhivotovsky LA Estimating population structure in diploids with multilocus dominant DNA markers Mol Ecol 1999 8 907 913 10.1046/j.1365-294x.1999.00620.x 10434412

[B82] Lynch M Milligan BG Analysis of population genetic structure with RAPD markers Mol Ecol 1994 3 91 99 10.1111/j.1365-294X.1994.tb00109.x 8019690

[B83] Vekemans X Beauwens T Lemaire M Roldán-Ruíz I Data from amplified fragment length polymorphism (AFLP) markers show indication of size homoplasy and of a relationship between degree of homoplasy and fragment size Mol Ecol 2002 11 139 151 10.1046/j.0962-1083.2001.01415.x 11903911

[B84] Peakall R Smouse PE Genalex 6: genetic analysis in excel. Population genetic software for research and teaching Mol Ecol Notes 2006 6 288 295 10.1111/j.1471-8286.2005.01155.x

[B85] Orloci L Multivariate analysis in vegetation research 1978 The Hague: Dr W. Junk B. V

[B86] Pritchard JK Stephens M Donnelly P Inference of population structure using multilocus genotype data Genetics 2000 155 945 959 10835412 10.1093/genetics/155.2.945 PMC1461096

[B87] Hubisz MJ Falush D Stephens M Pritchard JK Inferring weak population structure with the assistance of sample group information Mol Ecol Resour 2009 9 1322 1332 10.1111/j.1755-0998.2009.02591.x 21564903 PMC3518025

[B88] Evanno G Regnaut S Goudet J Detecting the number of clusters of individuals using the software STRUCTURE: a simulation study Mol Ecol 2005 14 2611 2620 10.1111/j.1365-294X.2005.02553.x 15969739

[B89] Earl D vonHoldt BM Structure Harvester: a website and program for visualizing structure output and implementing the Evanno method Conserv Gen Resour 2012 4 359 361 10.1007/s12686-011-9548-7

