# Supplementary material for: Multilocus phylogeography of the common lizard Zootoca vivipara at the Ibero-Pyrenean suture zone reveals lowland barriers and high-elevation introgression
Source: BMC Evol Biol. 2013 Sep 10;13:192. doi: 10.1186/1471-2148-13-192 (PMC3847509; doi:10.1186/1471-2148-13-192)

**Additional file 1**

*Multilocus phylogeography of the common lizard Zootoca vivipara at the Ibero-Pyrenean suture zone reveals lowland barriers and high-elevation introgression*

Borja Milá, Yann Surget-Groba, Benoît Heulin, Alberto Gosá, Patrick S. Fitze

**Supplementary Methods:** MtDNA amplification details: For both cyt-*b* and ND2 fragments, PCR mixes for 25-ul reactions contained: 2.5 ul of 5X Promega GoTaq Buffer, 1.5 ul of 10 mM MgCl_2_, 0.5 ul of 10 mM dNTPs, 0.5 ul of each primer (10 uM), 1 unit of Promega GoTaq polymerase, 2 ul of DNA template (10-150 ng/ul), and 17.3 ul of water. PCR cycles were as follows: 5-min denaturation at 94°C followed by 36 cycles of 94°C for 30 s, 53°C for 30 s, and 72°C for 90 s, with a final extension of 5 min at 72 °C. Products were purified with an ethanol precipitation and the cycle sequencing reaction prior to amplification was carried out with the forward amplification primer for each marker.

**Additional file 1: Table S1.** GenBank accessions for each *Zootoca vivipara* mtDNA haplotype used in the study.

|  | | | | |  | |  |
| --- | --- | --- | --- | --- | --- | --- | --- |
| **Concatenated mtDNA haplotype (ND2+CB)** | **ND2 haplotype** | **Genbank Accession** | **CB haplotype** | **Genbank Accession** | |  |  |
| **AA** | A | KF593874 | A | KF593892 | |  |  |
| **AB** | A | KF593874 | B | KF593893 | |  |  |
| **AD** | A | KF593874 | D | KF593895 | |  |  |
| **AE** | A | KF593874 | E | KF593896 | |  |  |
| **BC** | B | KF593873 | C | KF593894 | |  |  |
| **CC** | C | KF593870 | C | KF593894 | |  |  |
| **DA** | D | KF593877 | A | KF593892 | |  |  |
| **EA** | E | KF593867 | A | KF593892 | |  |  |
| **FH** | F | KF593866 | H | KF593899 | |  |  |
| **GG** | G | KF593876 | G | KF593898 | |  |  |
| **GH** | G | KF593876 | H | KF593899 | |  |  |
| **HH** | H | KF593882 | H | KF593899 | |  |  |
| **IF** | I | KF593879 | F | KF593897 | |  |  |
| **JH** | J | KF593878 | H | KF593899 | |  |  |
| **KH** | K | KF593871 | H | KF593899 | |  |  |
| **L1I** | L1 | KF593872 | I | KF593900 | |  |  |
| **LI** | L | KF593875 | I | KF593900 | |  |  |
| **MI** | M | KF593869 | I | KF593900 | |  |  |
| **NI** | N | KF593868 | I | KF593900 | |  |  |
| **PM** | P | KF593862 | M | KF593904 | |  |  |
| **QM** | Q | KF593891 | M | KF593904 | |  |  |
| **RL** | R | KF593888 | L | KF593903 | |  |  |
| **S1L** | S1 | KF593887 | L | KF593903 | |  |  |
| **SL** | S | KF593886 | L | KF593903 | |  |  |
| **T1L** | T1 | KF593880 | L | KF593903 | |  |  |
| **TK** | T | KF593881 | K | KF593902 | |  |  |
| **TL** | T | KF593881 | L | KF593903 | |  |  |
| **UM** | U | KF593863 | M | KF593904 | |  |  |
| **WL** | W | KF593865 | L | KF593903 | |  |  |
| **X1J** | X1 | KF593864 | J | KF593901 | |  |  |
| **XJ** | X | KF593889 | J | KF593901 | |  |  |
| **YL** | Y | KF593890 | L | KF593903 | |  |  |
| **ZN** | Z | KF593885 | N | KF593905 | |  |  |
| **VP** | V | KF593883 | P | KF593907 | |  |  |
| **V1Q** | V1 | KF593884 | Q | KF593908 | |  |  |

**Additional file 1: Figure S1**. Plots generated by the software Structure Harvester to determine the optimal K in the program Structure 3.1. (A) Delta K values from the method by Evanno et al. (2005). (B) Mean values of the estimated Ln probability of different K values.

A.

B.

**Additional file 1: Figure S2.** Results from an outlier analysis to detect loci under selection among 34 AFLP loci using the program BayeScan 2.0. Plots show Fst vs. posterior odds for selection for each locus in three population comparisons: (A) K = 2, (B) K = 3, and (C) K = 4. The vertical line in each plot indicates the threshold leading to a false discovery rate (FDR) of no more than 5%. Loci to the right of the line deviate from neutrality for the comparison shown. The loci represented by the dots sitting on the vertical lines are locus “15c194” in (A) and locus “19c84” in (B) and (C).

A.


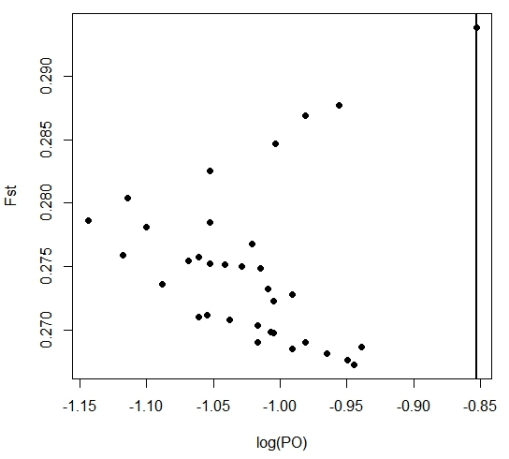


B.


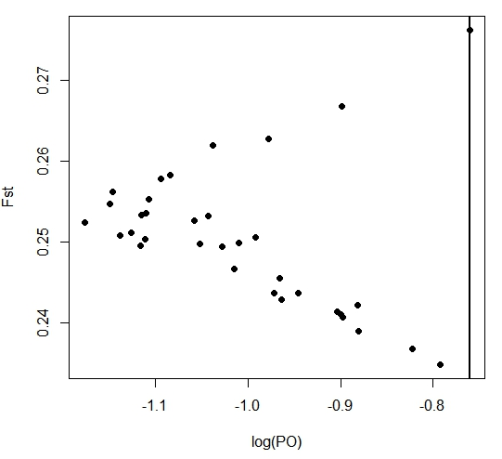


C.


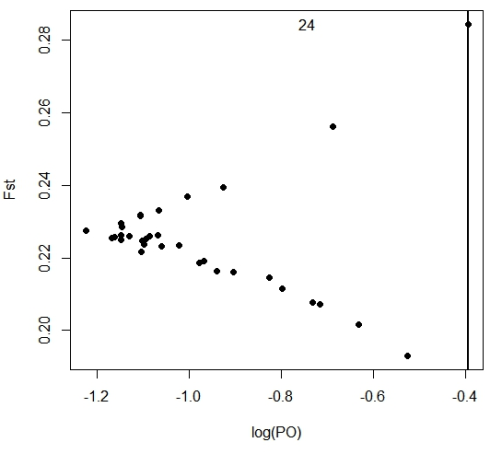

Supplement: Additional file 1: Table S1 — GenBank accessions for each Zootoca vivipara mtDNA haplotype used in the study. Figure S1. Plots generated by the software Structure Harvester to determine the optimal K in the program Structure 3.1. (A) Delta K values from the method by Evanno et al. (2005). (B) Mean values of the estimated Ln probability of different K values. Figure S2. Results from an outlier analysis to detect loci under selection among 34 AFLP loci using the program BayeScan 2.0. Plots show Fst vs. posterior odds for selection for each locus in three population comparisons: (A) K = 2, (B) K = 3, and (C) K = 4. The vertical line in each plot indicates the threshold leading to a false discovery rate (FDR) of no more than 5%. Loci to the right of the line deviate from neutrality for the comparison shown. The loci represented by the dots sitting on the vertical lines are locus “15c194” in (A) and locus “19c84” in (B) and (C). [file 1471-2148-13-192-S1.docx]
